# Supplementary material for: Safety and Survival Outcomes of Liver Resection following Triple Combination Conversion Therapy for Initially Unresectable Hepatocellular Carcinoma
Source: Cancers (Basel). 2023 Dec 17;15(24):5878. doi: 10.3390/cancers15245878 (PMC10741919; doi:10.3390/cancers15245878)

Supplementary figure S1. The standardized mean difference before and after propensity score analysis.

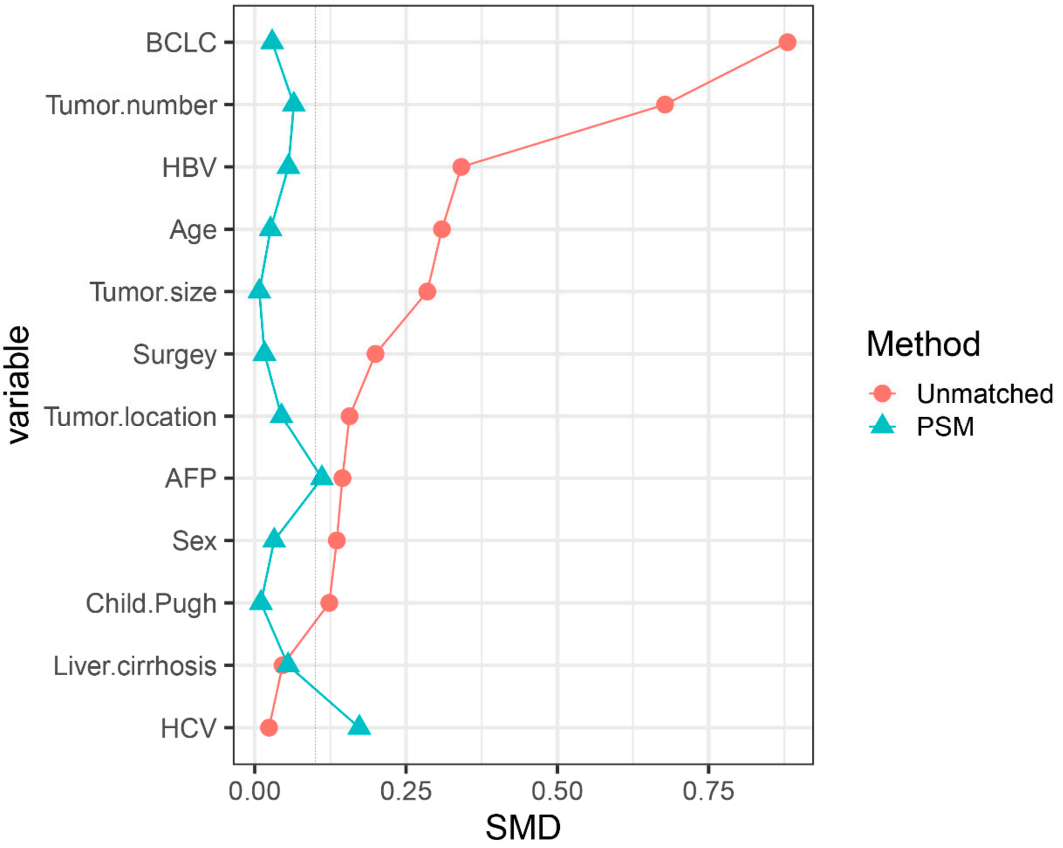

Supplement: Supplementary file 1 [file cancers-15-05878-s001.zip › cancers-2695455-supplementary.pdf]
